# Putting patients first: how risk awareness drives action among healthcare professionals in Slovakia

**DOI:** 10.3389/fpsyg.2026.1874950

**Published:** 2026-07-20

**Authors:** Lucia Kupkovicova, Andrea Madarasova Geckova, Zuzana Dankulincova Veselska, Radomir Masaryk

**Affiliations:** 1 Department of Psychology, Faculty of Arts, Comenius University Bratislava, Bratislava, Slovakia; 2 Department of Health Psychology and Research Methodology, Faculty of Medicine, Pavol Jozef Safarik University, Košice, Slovakia; 3 Faculty of Medical Sciences, The University of Groningen, Groningen, Netherlands; 4 Olomouc University Social Health Institute, Palacky University in Olomouc, Olomouc, Czechia

**Keywords:** healthcare professionals, hospital setting, patient safety, patient safety concerns, qualitative research, speaking up

## Abstract

**Introduction:**

The present study aimed to explore how healthcare professionals in Slovak hospitals perceive patient safety concerns and how these perceptions shape their actions.

**Methods:**

The study employed a qualitative research design using individual semi-structured interviews. The data were analysed using reflexive thematic analysis.

**Results:**

A total of 32 healthcare professionals with prior or current work experience in various departments of Slovak hospitals participated. The authors identified two themes: (1) patient health and safety as a core professional value closely linked to emotional experiences and fear of professional failure, and (2) context-dependent decision-making in response to patient safety concerns, with internal conflict when witnessing suboptimal care and efforts to protect patients through speaking up, intervening, or taking preventive measures.

**Discussion:**

The findings indicated that healthcare professionals conceptualised patient safety concerns not only as discrete patient safety issues, but also in relation to general health concerns, with a focus on ensuring safe and appropriate care. However, barriers to providing high-quality healthcare were also present, highlighting opportunities to improve safety culture and promote teamwork, shared responsibility, and open communication within hospital teams in Slovakia. The findings of this study may have relevance beyond the Slovak context, particularly in hospital settings in other post-socialist countries.

## 1 Introduction

Patient safety is defined as ‘a framework of organized activities that creates cultures, processes, procedures, behaviours, technologies and environments in health care that consistently and sustainably lower risks, reduce the occurrence of avoidable harm, make errors less likely and reduce the impact of harm when it does occur’ (WHO, 2021, p. 1). It has become a priority worldwide, resulting in the development and implementation of various patient safety strategies in healthcare (e.g., NHS England and NHS Improvement, 2019). In 2021, the WHO introduced the Global Patient Safety Action Plan 2021–2030 with the aim of reducing the number of preventable incidents that result in patient harm worldwide. Subsequently, in 2022, the WHO conducted a patient safety survey among Member States to assess progress in the initiative (WHO, 2024). The findings showed that, of the 108 countries that participated in the survey, 29% had developed a national action plan for patient safety, 38% had implemented a reporting system of never events, and 20% had integrated patient safety curricula into healthcare professionals‘(HCPs) education (WHO, 2024). The Ministry of Health of the Slovak Republic (2019) issued the Decree on the minimum requirements for an internal patient safety assessment system, which entered into force in January 2020 and includes the assessment of 13 different safety areas in institutional healthcare (e.g., safe patient identification, elimination, and prevention of falls). However, 5 years have passed, and there are only very limited data on the implementation of the decree in practice. For example, Hulková (2024) in her study critically pointed out non-compliance with the provisions of the decree regarding safe communication with patients in selected institutional facilities. Implementation gaps, such as those outlined above, imply that improving patient safety in practice depends not only on global and national policies and strategies but also on the culture within healthcare organisations and the broader cultural and historical context in which they are implemented.

According to WHO (2021, 2024), creating a safety culture based on a just culture instead of a blame culture is one of the key components of efforts to improve patient safety. Safety culture is a multidimensional construct that encompasses organisational, team, and individual factors that ensure patient safety, such as reporting systems and available resources, leadership commitment to safety, learning from mistakes and improvement, effective teamwork among staff and speaking up, awareness of human limits, and psychosocial factors (Churruca et al., 2021). The comparative study showed that safety culture differs across various European countries, highlighting several shortcomings that may be influenced by cultural and historical contexts (Granel-Giménez et al., 2022). Specifically, in Central and Eastern European countries (such as Hungary or Slovakia), safety culture may be shaped by medical tradition, in which healthcare environments have historically been influenced by strong hierarchical structures and paternalistic norms (Granel-Giménez et al., 2022; Murgic et al., 2015).

Safety culture in healthcare is associated with speaking up about patient safety or providing suggestions for improvement in healthcare practice (Lee S. E. et al., 2023; Rainer and Schneider, 2020). Research indicates that HCPs across various healthcare departments frequently encounter situations in which patient safety may be compromised because of healthcare delivery (Maxfield et al., 2013; Niederhauser and Schwappach, 2022; Schwappach and Niederhauser, 2019). Moreover, they are often able to recognise cues indicating that a colleague might be engaging in unsafe practice (Blair et al., 2022; Maxfield et al., 2005). Therefore, speaking up is an important communication strategy to alert others to potential patient safety risks (Schwappach and Niederhauser, 2019). The common motivation to speak up or intervene in these situations is the perception of a high risk of harm to the patient and the effort to protect them from harm (Blair et al., 2022; Lee et al., 2022; Okuyama et al., 2014; Schwappach and Gehring, 2014a). Although the decision to speak up may appear simple and linear, it is a complex consideration of various aspects of the situation, including the potential risks to the HCPs themselves (Blair et al., 2022; Schwappach and Gehring, 2014a). Under certain circumstances, these risks can lead them to withhold their patient safety concerns (Manapragada and Bruk-Lee, 2016). Hierarchical structures are a common reason for withholding in many countries across the United States, Europe and Asia (Lee et al., 2022; Schwappach and Gehring, 2014a; van Dongen et al., 2024).

Additionally, witnessing that patients do not receive adequate care from other HCPs is a common cause of moral distress (Eder et al., 2025; Henrich et al., 2016). Moral distress, originally described in relation to nurses as the inability to act ‘according to their own professional judgement and/or personal values due to external constraints or internal characteristics’ (Deschenes et al., 2020, p. 18), is also recognised among HCPs across various professional groups (e.g., Eder et al., 2025; Henrich et al., 2016; Lamiani et al., 2017). Another common problem causing moral distress is the lack of management support in providing healthcare and the lack of resources that can jeopardise patients (Granel-Giménez et al., 2022; Henrich et al., 2016). Research has also indicated that when nurses are unable to speak up about concerns at their workplace, their moral distress tends to increase, however, in strong safety cultures, they experience less moral distress (Rainer and Schneider, 2020).

Global and national strategies and policies form the foundation for prioritising patient safety in healthcare delivery (e.g., WHO, 2021), with a strong safety culture in hospitals being essential for fostering speaking up about patient safety concerns in daily practice (Rainer and Schneider, 2020). However, the recent study in Slovak hospitals showed that nurses perceived a low level of patient safety climate, with the most negatively rated aspects being non-punitive response to error, staffing, and teamwork across units (Gurková et al., 2020), which may negatively affect speaking up for patient safety. This qualitative study aimed to explore how healthcare professionals in Slovakia perceive patient safety concerns and how these perceptions shape their subsequent actions. The study has substantial potential to enhance understanding of contextual aspects related to patient safety concerns in Slovakia and may also be highly relevant to other under-researched Central European countries with post-socialistic tradition. Furthermore, the findings may help identify areas within healthcare delivery that require improvement.

## 2 Methods

### 2.1 Design

The present study is part of the broader research project focused on the perceived psychological safety of HCPs when speaking up about patient safety concerns in a hospital environment. The project was conducted using qualitative design and in-depth individual interviews. The study was reported in line with the Consolidated Criteria for Reporting Qualitative Research (COREQ) guidelines (Tong et al., 2007).

### 2.2 Sample selection and recruitment

The target population comprised HCPs working in hospital settings with at least one year of professional experience. Within this population, variation in sociodemographic and professional characteristics (e.g., age, position, and speciality) was sought in order to capture a range of perspectives and experiences.

Sample size was guided by the concept of information power proposed by Malterud et al. (2016). A relatively larger sample was considered appropriate given the broad aim of the study, the absence of a specific substantive theoretical framework, and the use of cross-case analysis to identify shared patterns of meaning across participants. At the same time, information power was strengthened by the high quality of the in-depth interviews and the relatively specific nature of the study population.

Participants were recruited from various hospitals in Slovakia using purposive and snowball sampling methods. Due to the use of snowball sampling across multiple recruitment channels, it was not possible to determine the exact number of HCPs who were approached. The study included HCPs working in a Slovak hospital at the time of data collection or working in a Slovak hospital for at least 1 year before data collection.

Prospective participants who met the inclusion criteria were approached via email, telephone or social media platforms and provided with essential information about the research project. Most HCPs agreed to participate, following which an interview was conducted. However, some of the approached HCPs did not respond, and a small proportion declined to participate due to time constraints. Others agreed to take part in the study, but a mutually convenient interview time could not be arranged, consequently, the interviews were not conducted.

### 2.3 Procedure

Data collection was carried out from April 2024 to March 2025 using individual in-depth semi-structured interviews. Most interviews were conducted in person at participant-selected locations (e.g., workplace or university) (*n* = 30). Two interviews (*n* = 2) were conducted online only when in-person interviews were not possible. Except for one interview, all interviews were conducted by the first author, a PhD student with prior experience in qualitative research. The remaining interview was conducted by another PhD student external to the research team due to availability constraints, which also allowed for reflection on whether interviewer variation might influence the interview process.

An interview was scheduled in the shortest time possible after the participant expressed an interest in participating in the study. Where feasible, an informed consent document was sent to them before any data collection. Shortly before the face-to-face interviews, participants signed an informed consent document. For online interviews, verbal consent was obtained at the beginning of the interview and written informed consent was provided subsequently.

Sociodemographic information about the participants was collected, specifically gender, age, highest level of education achieved, current position, current tenure of healthcare practice, current healthcare department, and the type of hospital in which they were employed.

A pilot interview was conducted with one head nurse to ensure the comprehensibility of the interview questions and assess whether they generated rich and relevant data. The pilot interview enabled the identification of questions for which alternative wording and interview prompts were required. The pilot interview was included in the final analysis for several reasons. First, the interview was of high quality in terms of its relevance to the research question, which Braun and Clarke (2022) identify as a key criterion in reflexive thematic analysis. In addition, the interview guide was not substantially revised following the pilot, and the participant inclusion criteria remained unchanged. The distinction between the pilot interview and subsequent interviews was therefore fluid, with the process better understood as a continuation of data collection. From a temporal perspective, there was a two-month gap between the pilot and the subsequent interviews, which was primarily due to the difficulty in recruiting participants. Furthermore, it was considered that the pilot participant was a head nurse employed in a private hospital, a subgroup that was particularly challenging to recruit.

### 2.4 Interview process

The average interview duration was 37 min. The interviews were audio-recorded, transcribed verbatim, and pseudonymised.

The interview guide consisted of 11 open-ended questions on three thematic areas: (1) factors influencing psychological safety when speaking about patient safety concerns; (2) strategies for speaking up about patient safety concerns; and (3) suggestions for strengthening psychological safety to support speaking up about patient safety. Some of the main questions were supplemented with sub-questions. The interview guide is included in the Appendix.

The interview questions were inspired by Morrison’s (2014) framework on employee voice and silence, and previous qualitative research studies that focused on psychological safety and speaking up in the clinical setting (e.g., O'Donovan and McAuliffe, 2020; Remtulla et al., 2021; Schwappach and Gehring, 2014b). An example of an interview question is “What do you consider in situations when you are concerned about patient safety?”

### 2.5 Ethical aspects and considerations

This study was approved by the Ethics Committee of Faculty of Social and Economic Sciences Comenius University (FSEV 161–2/2024) and was conducted in accordance with ethical standards, the principles of the Declaration of Helsinki, and the guidelines of the American Psychological Association.

All participants provided written informed consent. Through informed consent, the participants were informed about the aim of the research, course, and voluntary nature of their participation. Participants were also given the opportunity to ask additional questions before proceeding. They then provided written informed consent to participate, record the interview, and process their responses for scientific purposes. They were assured that they could withdraw from the research at any time without providing a reason and without any subsequent consequences.

Participants’ personal data were processed in accordance with GDPR requirements. During the interview transcription process, each interview was marked with a random numerical code that served as an identification code (pseudonymisation), and any parts of the text that could have led to the identification of the participants were removed from the text. Only the first author had access to the personal data of the participants and was able to decode their identities, if necessary. The audio recordings were stored in a secure repository that only the research team had access to.

### 2.6 Data analysis

The data analysis was conducted using various methods and approaches. The entire dataset was initially analysed using reflexive thematic analysis (RTA) as described by Braun and Clarke (2022), specifically limited to the first two phases: familiarisation and coding. The rationale for this choice was to analyse the perceptions and experiences of a heterogeneous research sample of HCPs across the entire dataset, which would not be possible with a similar type of analysis, such as interpretive phenomenological analysis (Braun and Clarke, 2021).

The analysis was underpinned by a constructivist epistemological position, which assumes that meanings are constructed through language and social interaction (Braun and Clarke, 2022). In line with this perspective, the analysis focused on how participants constructed meanings related to patient safety concerns within their workplace.

The familiarisation phase began during data collection, followed by repeated immersions in the collected data, both in the audio recordings during the transcription process and in the transcripts themselves. The authors actively and critically engaged with the data by asking questions to better understand their content.

The second phase involved abductive coding. This approach combined initial inductive coding to capture the meanings expressed by participants, followed by a later theoretical interpretation shaped by relevant theoretical models and research studies. Coding was conducted collaboratively by the first and second authors (a PhD student and a senior researcher) and in several sets. Each set of data was initially coded independently, followed by the collaborative discussion and further engagement with the data, thereby fostering reflexive interpretation in line with RTA. These collaborative discussions facilitated the exploration of additional meanings in the data and the refinement of codes. Through this iterative process, the entire dataset was systematically coded. The coding process was initially carried out manually on paper and later transferred to MAXQDA software for Microsoft (version 20) to facilitate qualitative analysis.

During the coding phase, one preliminary code (concerns about patient safety) stood out for its importance in the participants’ statements, appearing in more than two-thirds of the interviews. This code initially included specific situations in which HCPs expressed concerns about patient safety or patient health, for example describing what triggered these concerns, how they experienced them, and what they did in those situations. As this code appeared to represent a core aspect of the speaking-up phenomenon, a partial analysis of the text segments assigned to it was performed to provide a more nuanced and detailed interpretation. Thus, the remainder of the analysis presented here refers only to this subcorpus.

The authors extracted a subcorpus with segments for aforementioned code and proceeded with the reflexive thematic analysis by Braun and Clarke (2022). Given the conceptual breadth of the code, it was further refined into smaller analytical units to enable a more detailed exploration of its meaning. This was achieved through iterative coding, which involved the refinement and subdivision of the initial code into more specific subcodes. The codes were subsequently organised into candidate themes and subthemes according to patterns of shared meaning. The initial structure of the themes and subthemes covered cognitive (patient safety is a value), emotional (HCPs care about patients), and conative (HCPs act for the benefit of patients) aspects. A preliminary model of the themes, subthemes, and codes was created to facilitate the next phase. Notably, the analysis involved revisiting earlier phases when necessary, reflecting the iterative nature of the RTA analytic process (Braun and Clarke, 2022).

During the initial coding phase, the analysis focused on capturing semantic meanings in the data, whereas theme development was oriented towards identifying latent meanings. Consequently, the analysis combined semantic and latent levels of interpretation and was conducted within a constructivist framework. A reflexive journal was maintained throughout the process, from data familiarisation to theme development. It was used both to monitor subjectivity and to support ongoing analytic reflection. Notes were recorded on possible relationships between codes, recurring patterns, and potential interpretations. These memos were used to track the analytic process and to document the rationale for merging, splitting, or discarding certain themes.

During the review of themes phase, it became apparent that the initial thematic structure lacked a clear central organising concept, prompting revision of the themes. Consequently, a new structure consisting of two themes and six subthemes was developed and collaboratively re-reviewed with the third author. At this stage, the themes were still relatively broad and captured the overarching patterns that HCPs deeply cared about patients and quality of care and that they sought to protect patients’ health and safety. This was followed by a collaborative analytic discussion among the first three authors, which resulted in revisions to the thematic structure through the interpretative repositioning of several subthemes and codes. Thematic sufficiency was assessed in relation to the research question, with attention to distinctions within each theme.

In the final two phases of the analysis, the themes and subthemes were defined, their final titles were refined, a final thematic map was developed, and the analysis was reported in this manuscript. The selected participant quotations were translated into English and underwent minor editorial adjustments to enhance clarity and readability while preserving the participants’ original meaning.

### 2.7 Reflexivity and positionality of the researchers

Conducting reflective thematic analysis required a reflective process and recognition of researchers’ positionalities.

The study (including its design, data collection, and analysis) was conducted by a team consisting of a PhD student and three researchers (psychology professors), all of whom are authors of the present study. A research assistant, who was a PhD student at the time of data collection and is currently a researcher, assisted with data collection.

In terms of the authors’ professional positionalities, the entire team consisted of psychologists, which informed a strong psychological perspective on the topic. In this sense, the authors were outsider researchers, however their diverse research experiences with patients, HCPs, or both, enabled a sensitive understanding of both perspectives and, in this study, facilitated engagement with HCPs. The second and third authors had long-term interdisciplinary research experience and were in intensive contact with HCPs and patients. The third and fourth authors supervised the qualitative research process, while the last author contributed a perspective grounded in social psychology, enriching the analysis with additional insights. These professional positionalities shaped the study design, data collection, analysis, and reporting of the results.

All interviews were conducted by two PhD students in psychology with interviews being conducted by a single interviewer at a time. Both had backgrounds in psychology and prior experience in qualitative research. Thirty-one interviews were conducted by the first author of the study who became familiar with the research topic prior to data collection and who, as part of her master’s degree, conducted similar qualitative research focused on the experiences of HCPs with patient safety incidents. Based on her prior experience, the first author anticipated potential interview topics (e.g., medical hierarchy). She avoided introducing them directly but pursued them when they arose, which may have led to some topics receiving more attention than others. From a personal perspective, the first author was particularly interested in experiences of psychological safety when speaking up and at times found it difficult to avoid associating her own experiences with those of the participants. Additionally, one of the interviews was conducted by another PhD student at the time of data collection, a psychologist graduate with nursing training who had extensive experience conducting narrative interviews. The background of the PhD student might have influenced the data collection in the interview, with extended empathy and knowledge regarding the healthcare system in Slovakia.

During the interviews, power dynamics between interviewers and participants may have influenced the interview process in different ways. As psychology PhD students, the interviewers may, at times, have perceived HCPs with senior positions and extensive clinical experience as figures of authority, potentially shaping how certain topics were explored during the interviews. At the same time, participants may have perceived the interviewers as relatively low risk interlocutors due to their position outside hospitals, which may have facilitated openness when discussing sensitive experiences. Furthermore, participants may have provided more detailed explanations of clinical practices and workplace contexts, recognising that the interviewers did not share their professional background.

Reflexivity was facilitated throughout the analytic process through regular collaborative discussions among members of the research team, which provided opportunities to share interpretations and explore understandings of the data. In addition, reflexive engagement occurred individually, with researchers reflecting on coding decisions and theme development and documenting these reflections through analytic notes. For example, during the initial stages of theme development, codes were tentatively organised according to cognitive, emotional, and behavioural components. However, following ongoing reflection, discussion, and further engagement with the data, it was concluded that this structure did not adequately represent participants’ accounts. Instead, it appeared to reflect authors’ own background in psychology more than organising patterns from the data. Recognising this, the former structure was abandoned, and new candidate themes were developed. A key contribution to this stage of the analysis came from the third author, who approached the data with a different analytical lens informed by her experience in qualitative research. This contributed to a decision to further reorganise the new structure of themes and subthemes considering newly identified meanings in relation to the main organising concepts. This process led, for example, to the reconceptualisation of the subtheme *Experiencing concern and internal conflict in response to suboptimal care*, which was reframed as an aspect of a broader theme.

## 3 Results

### 3.1 Research sample

A more detailed description of the sample is provided in Table 1 (Sample characteristics). The entire sample consisted of 32 healthcare professionals with prior or current work experience in Slovak hospitals, collectively representing 19 different healthcare departments (e.g., neonatal intensive care medicine, internal medicine). The ages of the participants ranged from 25 to 58 years, with a mean age of 39.2 years (SD = 11.7). The duration of clinical practice ranged from 1 year and 5 months to 39 years. The median duration of clinical practice was 10 years (IQR = 24.75). Most participants were employed at a state hospital (*n* = 25).

**Table 1:** Sample characteristics (*n* = 32).

Characteristic	Category	n
Gender	Female	22
	Male	10
Education	Upper secondary vocational education	3
	Post-secondary vocational education (Diploma in Nursing)	1
	Bachelor’s degree	5
	Master’s degree	17
	Doctoral degree	6
Hospital department	Sports diagnostics, traumatology, and orthopaedics	1
	Neonatal intensive care medicine	4
	Neonatal department	1
	Geriatrics	1
	Internal medicine	3
	Surgery (Intensive Care Unit)	2
	Gynaecology and obstetrics	2
	Anaesthesiology and intensive care medicine	1
	Internal gastroenterology	2
	Paediatric oncology and hematology	1
	Neurology	4
	Dentistry and maxillofacial surgery	1
	Vascular Surgery	2
	Radiology	1
	Psychiatry	1
	Urology	1
	Rehabilitation	1
	Angiology	1
	Arrhythmia department	1
	Emergency department	1
Hospital type	State	25
	Private	3
	A joint-stock company	4

The sample included 12 physicians, 10 nurses, 1 head physician, 1 head of the clinic, 3 head nurses, 4 day-shift nurse managers, and 1 physiotherapist. Table 2 presents the distribution of gender across these positions.

**Table 2:** Gender distribution by position (*n* = 32).

Position	Male (n)	Female (n)	Total (n)
Physician	6	6	12
Nurse	3	7	10
Head physician	0	1	1
Head of the clinic	1	0	1
Head Nurse	0	3	3
Day-shift nurse manager	0	4	4
Physiotherapist	0	1	1

Although the sample comprised 32 participants, the phenomenon under investigation in this study was explicitly discussed in 26 interviews. The fact that this phenomenon did not explicitly emerge in the remaining six interviews may have been influenced by the specific nature of managerial roles, as 4 of these interviews were conducted with nurses in managerial or leadership positions, with the remaining participants being 1 nurse and 1 physician, although alternative explanations cannot be excluded.

### 3.2 Themes, aspect of the theme and subthemes

**Figure 1:**
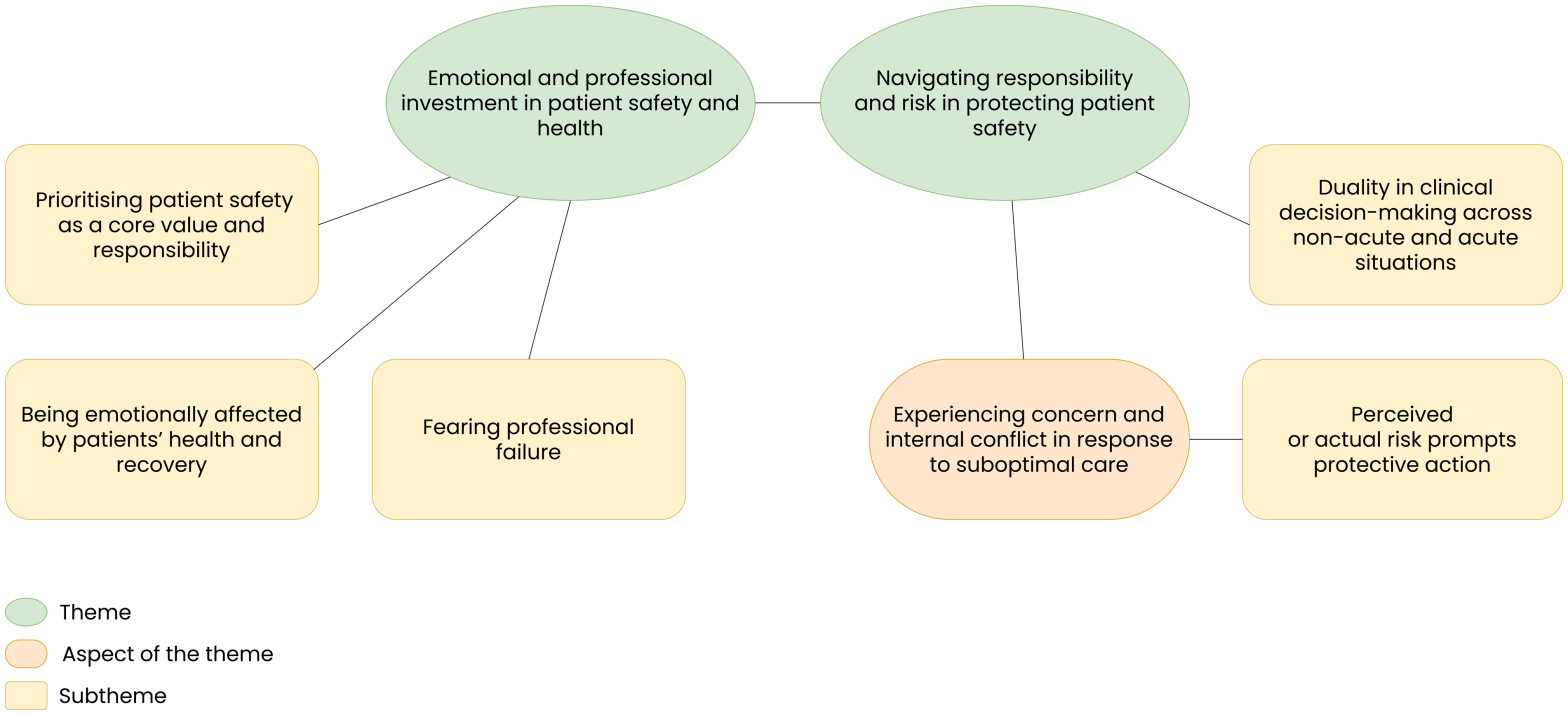
Visual representation of the identified themes, an aspect of the theme, and subthemes.

The authors processed the results of the analysis at three levels: (1) themes, (2) a specific aspect of the theme (an intermediate level between the theme and subtheme), and (3) subthemes. Two themes, one aspect of the theme and five subthemes were identified (Figure 1):

1. *Emotional and professional investment in patient safety and health* with three subthemes: (A) Prioritising patient safety as a core value and responsibility; (B) Being emotionally affected by patients’ health and recovery; and (C) Fearing professional failure;
2. *Navigating responsibility and risk in protecting patient safety* with two subthemes: (A) Duality in clinical decision-making across non-acute and acute situations; (B) Perceived or actual risk prompts protective action (with one aspect of the theme: Experiencing concern and internal conflict in response to suboptimal care).

It is important to emphasise that the interview guide was centred on patient safety issues, however, the interviews indicated that HCPs did not clearly distinguish between general concerns about patients’ health and patient safety but rather perceived them as closely interconnected. Accordingly, in this study, patient safety concerns were defined broadly as situations perceived by participants as posing a potential risk to patient safety, regardless of the underlying cause. For this reason, general concerns and patient safety concerns were not analytically separated but were instead treated as a single phenomenon. In other words, the aforementioned themes, an aspect of the theme, and subthemes relate to both general health concerns (e.g., concern about an unstable patient or deterioration in the patient’s condition) which may potentially result in patient safety issues, and to patient safety concerns (e.g., the occurrence of a patient safety incident, for example patient falls).

In presenting the findings, anonymised participant quotations are included. For contextual purposes, each quotation is accompanied by the participant’s position and seniority category: early-career (0–5 years of experience), mid-career (6–15 years of experience), or senior (16 + years of experience).

#### 3.2.1 Emotional and professional investment in patient safety and health

Participants described a range of situations in which they experienced concerns about patient safety, including those arising from inadequately provided healthcare that could lead or had led to patient safety incidents. Among these situations, participants mentioned patient falls, failure to administer medication or administering the wrong medication, incorrect diagnosis, and colleagues not using aseptic or protective barriers during medical procedures (e.g., gloves, gowns). However, some participants reported no experience of compromised patient safety in their hospital and instead described situations involving deterioration in a patient’s condition, regardless of the cause. In such cases, their focus was primarily on the patients’ clinical conditions and safeguarding their health, during which they sometimes experienced concerns about potential patient safety incidents. There were also instances in which despite prompting by the interviewer, participants were unable to frame concerns in terms of patient safety and instead described unrelated situations.

In this particular theme, HCPs demonstrated strong emotional and professional investment in patient safety and health, viewing it as their highest priority and core value. Because patient care is very important to them, they are emotionally affected by patients’ treatment outcomes and recovery. However, this commitment also places them in situations in which they face a natural fear of professional failure that could harm patients.

##### 3.2.1.1 Prioritising patient safety as a core value and responsibility

Participants emphasised that the patient is their highest priority and that they want to do everything in their power for them, ensuring their sense of safety and satisfaction with the healthcare provided. The way most participants expressed themselves suggested that this was their personal value.

*“I think it’s really important, because whether it’s a small patient or just any patient at all, it’s important that safety is maintained and that they feel safe.” (day-shift nurse manager, senior).*

Some HCPs reported that it was a value shared by the entire team that was related to effective teamwork. This included joint discussions on what would be best for the patient and teamwork when managing a patient’s medical condition.

*“We’ve really set ourselves up so that, first and foremost, the patient comes first – everything is for the patient.” (nurse, early-career).*

*“Our team is fairly open and collaborative, you know? The patient’s interests are the highest priority for everyone here – really the top priority – and that’s what we are all aiming for.” (physician, early-career).*

Perceiving the patient as a priority is not only related to personal or team values, but also to the aspect of professionalism and responsibility. Some participants explicitly stated that the fact that they work professionally as a physician or nurse does not allow them to underestimate the situation and makes it easier for them to speak up.

*“We work with people, so we have to talk about this openly, because if something were hidden – if a nurse hid the fact that a patient had fallen – it could even end fatally. […] From a professional standpoint, I have to communicate about it. My professionalism simply would not allow me to keep it from someone, because I’m taking on the risk myself, right?” (head nurse, senior).*

##### 3.2.1.2 Being emotionally affected by patients’ health and recovery

Since patient safety and health are perceived by HCPs as a value and professional responsibility, they experience fear and concern about patients’ health or their health condition. In this regard, some participants stated that it is an unpleasant situation for them, where they experience feelings of anxiety or stress because they do not know whether everything will turn out well, and the patient will be okay.

*“So basically, it’s fear – fear for the patient’s safety. You’re just worried about the patient, and it’s a very uncomfortable feeling. You cannot focus on anything else; all you think about is what could happen.” (nurse, mid-career).*

*“[…] you just have this natural concern, because people’s lives are in your hands—many lives, and even more so when you are on duty. So of course, you have that natural worry about whether you’ll be able to save everyone if their condition suddenly worsens or at least help them as much as possible.” (physician, early-career).*

Some participants reported that their emotional state depended on how the patient felt and what they were experiencing.

*“It’s also a kind of despair and helplessness. I want to help the patient – I have this patient in front of me for twelve hours, and I can see that they are suffering. It just does not sit well with me.” (nurse, early-career).*

##### 3.2.1.3 Fearing professional failure

Participants, predominantly nurses, reported feeling responsible for patients and for ensuring they were not exposed to risk during health care provision. The participants were fully aware of the consequences of their actions on the patients and were therefore afraid of causing harm to them. This reflects a fear of failing professionally and causing patient safety incidents. One nurse highlighted a tension between different medical and nursing priorities—saving the patient at all costs versus avoiding harm to the patient during the provision of care:

*“What I worry about most is that we do not harm the patient. Yes, that’s often the priority. In surgery, it’s common to keep going – sometimes people say operating ‘to the death’, pushing through even against the patient’s will. We try to save them at all costs. But from our nursing perspective, or from a caregiving point of view, our main concern is the fear of causing iatrogenic harm to the patient, more than the harm the patient might sometimes cause to themselves.” (nurse, mid-career).*

*“We were really aware of the mistakes that could happen. The staff recognised them too, but the problem was that actions were driven by emotions. When you realise the potentially fatal impact on a patient, your reaction can sometimes be exaggerated and emotional. It’s not shouting, but more indignation – people getting frustrated with each other. They’re aware of their responsibility, especially the nurse, because ultimately, she is responsible for the patient. Since I’m a nurse working with other nurses, it affects us negatively too, because we take it as a personal failure.” (nurse, mid-career).*

Some participants stated that they felt better and more comfortable when working with another HCP they could rely on to provide healthcare, for example, to prevent or resolve patient safety incidents or other unexpected events.

*“I always prefer to have a practical nurse with me, so that some kind of double-check can take place. And when there are two of us, I feel it’s a bit more relaxed […]” (nurse, early-career).*

#### 3.2.2 Navigating responsibility and risk in protecting patient safety

Within the context of perceived responsibility, HCPs respond to risk by adopting protective behaviour towards patients. They consider two pathways: (1) they carefully make decisions based on the type of situation (non-acute or acute), the risks and benefits for the patient, and the risks to themselves; and (2) they experience an internal conflict between the right course of action and what they can realistically do, which leads them to act.

##### 3.2.2.1 Duality in clinical decision-making across non-acute and acute situations

HCPs’ decision-making differs depending on the type of situation, namely non-acute and acute situations.

In non-acute situations, participants reported that their actions are sometimes preceded by a careful decision-making process, during which they consider the potential benefits and risks of a particular health decision or procedure to patients. For example, physician described the difficulty of deciding whether to transfer patients whose condition was not improving to a higher-level facility or keep them in their current setting, as neither option guaranteed a better outcome. While making this decision, she reflected on the fact that several physicians across previous shifts managed the same patient without opting for transfer. This required her to independently assess the risks and benefits of transferring the patient and come to terms with her decision internally, even if the situation was to develop unfavourably.

*“For us, a big question was always when to keep the patient on our ward. Not at the highest level in the hierarchy, but at what point the patient belongs at a higher level of care. It’s always a sensitive topic, because on one hand, I want to help the patient: the benefit of keeping them with us is a less invasive environment, lower bacterial exposure, and the presence of the mother on the ward. These are all benefits for the patient. On the other hand, I use up the therapeutic options more quickly.” (physician, mid-career).*

HCPs sometimes found themselves in a dilemma in which they perceived a risk to the patient that could potentially lead to harm, but at the same time, speaking up about it posed a risk to themselves. In such situations, HCPs rationally assessed the level of risk to the patient and compared it with the expected potential obstacles (whether to invest energy, provoke conflict, or disrupt relationships) associated with speaking up to resolve the issue.

*“Yes, first of all, it’s about whether it will harm the patient or not. And whether I have any control over it – whether I can influence it or not. I also consider the risk for myself, whether I might be putting myself at risk as well.” (physician, senior).*

High patient risk motivated them to address the situation, but in cases of low patient risk, some participants expressed that they were hesitant to respond or opt not to address the issue.

*“If I assess the situation and see that something serious could happen, something that might harm the patient, then I would probably speak up.” (nurse, senior).*

*“But when it’s milder, I hesitate whether it’s even worth mentioning. Whether to pursue it and invest not only my energy, but also the other person’s, or another team’s, into it, whether to just let it slide, or really go for it.” (physician, early-career).*

HCPs are also concerned about possible legal repercussions of their actions.

*“And now, if something does happen, you start thinking about possible legal consequences, what if there’s a serious incident, what if a problem arises, what if the patient complains. Of course, these thoughts go through my head – there’s no doubt about that.” (physician, early-career).*

However, in acute situations (e.g., acute bleeding), participants stated that it was necessary to act quickly and intervene to stabilise the patient or prevent harm, resulting in minimal room for lengthy decision-making and discussion.

*“[…] when there’s a resuscitation or an emergency situation, for example if the child somehow self-extubates or needs resuscitation, in those cases you do everything quickly. You do not look to the right or left, you just need to save the child.” (day-shift nurse manager, senior).*

*“When it’s acute care, when it’s a matter of life and death, sometimes there’s no time to talk much and you really have to act quickly. And you have to be very, very selective – not talking too much, just saying what’s necessary.” (physician, senior).*

##### 3.2.2.2 Experiencing concern and internal conflict in response to suboptimal care

HCPs reported various situations involving the provision of suboptimal healthcare. These situations caused an internal conflict between what they considered to be the right action and their inability to do so due to various factors. These situations were related to collaboration and communication among HCPs, the way healthcare was provided by other HCPs, hospitals’ established processes, or infrastructure.

Providing healthcare in a hospital often requires collaboration and communication between nurses and physicians or interdisciplinary collaboration between HCPs. Participants experienced negatively when they needed to address a patient’s condition, but ineffective teamwork and communication stood in their way and did not work as they expected, leading to a greater risk to patients and their suffering.

*“Or sometimes we have patients who are bleeding, every minute counts, and the emergency intake team is not able to take the blood samples. […] And then, fifteen minutes later, we have the patient in shock, and we have to draw the blood again, which is just unnecessary suffering for the patient […] We’ve already talked about it several times, that it should be done, but it still is not.” (nurse, mid-career).*

*“[…] when a consulting physician comes and leaves a patient waiting – as used to happen with the internists and the pulmonologists arguing over whether it’s pneumonia or heart failure, since one belongs to the pulmonologists and the other to the internists. They would argue over which case was more severe so that the patient would be sent to the other department. Sometimes that took eight hours, and a few times the patient actually died, because while they were arguing, the patient needed antibiotics but did not get any for eight hours, since no one administered them.” (physician, senior).*

*“This is exactly the point: the care itself is what suffers. I’m not saying this because I want the [name of the ward] to be punished or anything like that, but because in the end we are the ones who have to deal with the patient afterward. Their treatment gets prolonged, their condition worsens, they end up on unnecessary antibiotics and XY other things. So that’s the perspective, in the end, it’s about the patient again, but somehow that gets lost along the way.” (nurse, early-career).*

The issue with night shifts was a recurring situation, when a nurse or physician would call their colleagues to the patient but were repeatedly met with physicians’ reluctance to come and jointly address the patient’s health condition. Subsequently, the participants either attempted to manage the situation within their own capacity or prompted the physician to take action (either by coming in person or providing support remotely). The situations in which some HCPs were indifferent to the patient’s needs were associated with participants’ feelings of discomfort, embarrassment, or helplessness. In the following quote, a nurse highlighted that physicians often focus on individual medical procedures rather than on the patient’s overall holistic care within the hospital setting:

*“And this exact situation happened: the patient had undergone head surgery, and the wound on her head started bleeding. […] So, my colleagues called the physician to explain what was going on, and he hung up the phone without saying a single word. He did not come to check on the patient the whole night. […] And honestly, I get the feeling that the physicians just do not care, like, ‘I’ve done the surgery, and whatever happens afterward is not my problem.’.” (nurse, early-career).*

Some participants also expressed concerns when they felt that other HCPs did not follow standards or even neglected care, which could have or had a negative impact on the patient.

*“[…] I’ve had experiences with colleagues where, when a patient is in poor condition – for example, they might have trouble swallowing a pill or something like that – some of them just leave it and say, ‘I’ll let the next nurse handle it.’ And if the next one cannot manage either, she might give the medication anyway, even though there’s a risk something could happen. I’ve also seen cases where they simply record the medication as given, because, well, paper can handle anything, but the patient never actually receives it.” (nurse, early-career).*

*“If it had been properly documented and diagnosed at the time, because it was a life-threatening condition, they would have operated long ago. So, the patient would not have had to wait three days for the surgery. But by then, the patient was already in such poor condition that when they finally operated, he ended up in the ICU afterward, because he went into some kind of shock and so on.” (physician, senior).*

One participant pointed out that sometimes even the HCPs themselves are the ones who encourage less experienced or new employees not to follow some safety rules that could potentially endanger patients.

*“Sometimes the problem comes from the physicians’ side as well. For example, if a physician says, ‘Come on, let us go quickly, do not bother taking anything because I’m not taking anything,’ then the nurse does the same. And then we can try to, you know, talk to the nurses when the physician does not stick to it.” (day-shift nurse manager, senior).*

Another problem that HCPs perceived was poorly set processes (e.g., patient management, work organisation) and insufficient infrastructure in the hospital, which increased the risk of patient safety incidents. Participants perceived that their work could be more effective and safer for patients if it were structured to incorporate nurses’ and physicians’ insights from daily practice and better consider patients’ experiences in the hospital. Some participants mentioned pricking a patient’s vein multiple times during blood collection, which they perceived as unnecessary pain when other options existed (e.g., blood collection through a catheter).

*“But we really just want to make patient care easier. I’ll give a specific example. The patient needs to have blood drawn. On this ward, they are used to always sticking the patient, whereas a nurse in the [name of the ward] has other options, not just repeatedly puncturing the vein. […] And when we from the [name of the ward] explain that this is actually the easiest way and provides a precise sample, either from arterial blood or a central venous catheter, they simply do not want to do it. They only do it the old way, so as not to introduce ‘new habits’. But it’s not about introducing new habits, it’s about making it easier for the patient. Why make them suffer extra pain, while we can take the blood almost painlessly? I just turn the tap, draw the blood into the tube, and the patient will not even feel it. Yet they still do not want to do it this way.” (nurse, senior).*

*“If those factors, like the environment, for example, were better managed, for instance if we could improve the workflow between the outpatient clinic, the operating theatre, and the ward, we collaborate across all of these, then even improving just one factor could make a difference, even if it’s something that depends on multiple people. […] For example, the physicians would need to plan patient admissions differently, and we as nurses would need more time to prepare the patient for the operating theatre, because sometimes they call from the operating theatre saying they want the patient now, and the patient is not ready yet. And there’s also a bit of pressure to hurry, because they are already dressed and waiting in the operating theatre.” (nurse, early-career).*

Participants also expressed concerns about insufficient infrastructure, including insufficient equipment (e.g., non-functioning elevators, lack of disposable hygiene products) and insufficient infrastructure on hospital premises (e.g., a broken road, non-functioning outdoor lighting), which made it difficult to move patients in beds to other hospital buildings. For HCPs, this constitutes a challenge in the provision of care that lies beyond their individual capacity to improve, although they are still expected to deliver high-quality care under these conditions.

*“A perfect example: we have three lifts here, we are on the third floor, and two of them were not working. We were already joking that if a fire broke out, we’d have to go out the windows, or something like that, with the patients. Often it ends up being that kind of laughter out of despair: ‘what are we going to do?’, because it’s such a risk.” (nurse, mid-career).*

##### 3.2.2.3 Perceived or actual risk prompts protective action

HCPs described various situations in which they acted for the benefit of patients, with a common way of acting being speaking up. Most participants reported that when they became aware of the potential or actual risk of a patient safety incident that could result in patient harm or noticed that a patient’s condition had suddenly worsened, it motivated them to speak up about it, ask for advice, or consult with others.

Some participants described specific instances in which they directly alerted a colleague to a safety risk or witnessed an alert between colleagues. HCPs considered this particularly important because they perceived the potential impact on the patient (e.g., transmission of infection).

*“So, I naturally try to alert the colleague in question, or even the patient, that something like this could pose a risk. […] Of course, when it becomes apparent that something might go wrong, for example during a longer procedure or operation, if we notice that a colleague’s concentration is slipping or they are not being careful, we can point it out. And I think in this regard, we are quite helpful.” (physician, mid-career).*

Some participants tended to speak up regardless of potential negative consequences (e.g., expected reaction of the other party, disruption of relationships), while others adapted their communication style to the specific person they are going to communicate with in order not to offend someone, not to provoke conflict.

*“In the end, I always tell myself that the patient comes first. [.] So, in the end, I always communicate whenever something seems off, always, always, always.” (physician, early-career).*

*“[…] we generally try to communicate, yes. Although it’s challenging with them, because they often brush us off, saying things like, ‘We’re operating now, we do not have time,’ and so on.” (nurse, early-career).*

*“When it was a colleague with years of experience, communication with her was handled more delicately, but we always tried to make sure it did not come at the expense of the patient’s health.” (physician, mid-career).*

Participants also reported efforts to prevent situations in which a patient could be at risk - either before any patient safety incident occurred or because of it occurring. Examples included transferring an employee to a ward with less serious inpatient conditions, using side rails or restraints to prevent patient falls, preventive door locking, and employee retraining.

*“[…] if we have concerns about patient safety, we often take preventive measures, such as locking all accessible doors for the patients’ safety, even if we are not sure how the patient will react. For their protection, we always implement these measures as a precaution.” (physician, senior).*

The head physician described how patient safety incidents, when systematically addressed with relevant stakeholders, can lead to improvements in future care delivery:

*“[…] we had a situation where a patient was given a higher dose of medication than intended. The dose had been incorrectly prescribed, so we addressed it directly with the physician involved and communicated about it, but we also reported it externally to the hospital management as an extraordinary event. This was because the patient could have been at risk, even though fortunately there were no side effects. At the same time, we implemented measures ourselves to prevent this from happening in the future, because it was a very specific medication that we use very rarely, so we made sure there was a double-check system in place. In other words, we were looking for a solution to make sure it would not happen again.” (head physician, senior).*

One participant would especially appreciate it if the hospital staff were informed about the frequency and type of patient safety incidents and if measures were created to prevent them, which is not happening now.

*“In healthcare, or among us nurses, we talk about adverse events, medication errors, pressure ulcers, falls, and I do not even know what else. And you have no idea what actually happens in this hospital, how many patients have fallen, how many were seriously injured, how many pressure ulcers occurred, how many we caused ourselves, how many came from outside. It’s absolutely not working.” (nurse, mid-career).*

## 4 Discussion

The present study aimed to explore how HCPs in Slovakia, a Central European country, perceive patient safety concerns and how these perceptions shape their actions. The focus of the study was patient safety issues occurring in hospitals during patient care. Interviews with HCPs revealed that participants conceptualised patient safety concerns more broadly, with patient safety issues (e.g., patient falls, risk of medication errors) appearing closely intertwined with general patient health concerns. In this sense, participants tended to focus less on categorising concerns (general health vs. patient safety) and more on ensuring that patients remained safe and received appropriate care. This is a noteworthy finding, as it reflects the complexity of healthcare delivery and the ultimate goal of achieving safe and appropriate patient care outcomes.

Two main themes were identified in this study: (1) Emotional and professional investment in patient safety and health; and (2) Navigating responsibility and risk in protecting patient safety.

Within the first theme, the findings suggested that HCPs consider patients’ health and safety as the highest priority stemming from their values and sense of professionalism and responsibility. This theme also showed how participants’ values intersect with their emotional experiences, leaving them emotionally affected by patients’ experiences and fear of professional failure. This is in line with previous research highlighting the role of HCPs as patient advocates committed to ensuring both patient safety and making patients feel cared for and safe during healthcare delivery (Heier et al., 2021). Moreover, Okuyama et al. (2014) identified HCPs’ responsibility towards patients and their perception of their professional role as individual factors influencing speaking up about patient safety. In line with aforementioned, the present findings also indicate that HCPs predominantly perceive the protection of patient safety and health as their personal values and commitments. Although team value was rarely explicitly mentioned, participants likely perceived it as a shared responsibility within the team. In a qualitative study by Heier et al. (2021), HCPs acknowledged individual levels of safety performance, such as adherence to safety-related rules, but also emphasised the team level, which included teamwork and sharing knowledge.

The second theme captured the HCPs’ active efforts to provide safe healthcare and protect patient safety, even in demanding and complex situations. The results revealed a duality in participants’ decision-making, whereby non-acute and acute situations require different approaches for managing clinical situations. In non-acute situations, given time and space, HCPs carefully weigh the potential benefits and risks to the patient and act on their decisions. The non-urgent nature of the situation makes professionals’ decision making susceptible to various factors (e.g., individual, organisational), including clinical severity and perceived personal risks, which may be assessed consciously or unconsciously. Having patient safety concerns, perceiving the risk to patients and efforts to protect them often serve as primary motivators for speaking up (Blair et al., 2022; Grailey et al., 2021; Okuyama et al., 2014; Schwappach and Gehring, 2014a). Consistent with previous studies (Blair et al., 2022; Schwappach and Gehring, 2014a), some participants expressed concerns regarding the negative consequences of speaking up for themselves, which could be linked to low psychological safety within the workplace (Grailey et al., 2021). Conversely, in the event of an emergency or imminent risk to the patient, participants reported that they would take immediate and appropriate action, which has been reported in previous studies (Blair et al., 2022; Kupkovicova et al., 2024). This reflects that, in acute situations, individual, organisational, and other contextual factors are likely to become less salient, as HCPs focus on their primary task of saving the patient’s life.

A particularly strong aspect of this theme was the situation in which HCPs experienced internal conflict between witnessing suboptimal healthcare provided by colleagues and feeling unable to act in a way they deemed best for the patient. This often prompted them to act either by speaking up or intervening. In international literature, this is termed moral distress (Deschenes et al., 2020), although only one participant in the present study described the situation in these terms. In their study on moral distress, Morley et al. (2020) developed definitions for distinct types of moral events. The experiences described by participants are most closely aligned with what the authors refer to as moral conflict, which may manifest as frustration, anger, or helplessness (Morley et al., 2020). Although most participants did not explicitly label their emotions, their word choices and tone of voice indicated the presence of these affective states. Morley et al. (2020) also noted that moral conflict tends to be expressed externally, which corresponds with the findings of the present study that participants were motivated to speak up or intervene in the best interests of patients when faced with perceived or actual risks. Henrich et al. (2016) also demonstrated that witnessing inadequate care causes moral distress among HCPs. The present study supports these findings, including the inadequate timeliness of physicians’ responses to patients’ needs, exposure of patients to unnecessary pain, and resource constraints that impede the delivery of high-quality healthcare (Henrich et al., 2016).

This internal conflict further highlighted a power imbalance between physicians and nurses, which was reflected predominately in nurses’ reports that they were unable to rely on physicians as their requests for assistance were not given adequate attention. Hierarchical relationships between physicians and nurses and physicians’ responsibility for the final decision are common reasons why nurses often feel unheard, underappreciated, and disrespected by physicians (Henrich et al., 2016; Lokatt et al., 2023; Mawuena et al., 2024; Morley et al., 2020; Vos et al., 2025). Consequently, feeling undervalued within a team harms cooperation in healthcare delivery (Stadick, 2020). Given the historically entrenched paternalism in physician - patient relationships in Central and Eastern Europe (Murgic et al., 2015), it is possible that similar dynamics are reflected in physician - nurse interactions. In this context, physicians may be positioned as both formally and informally dominant in decision-making processes, potentially constraining nurses’ autonomy (Lee M. et al., 2023). An aspect of particular relevance is the crucial role of responsibility for the patient in hierarchical relationships between physicians and nurses. In the study by Mawuena et al. (2024), nurses perceived the physician as responsible for the patient and therefore decided not to speak up about patient safety concerns or propose solutions. However, in present study, nurses expressed a strong sense of responsibility for patients, which may motivated them to speak up or take action, even within hierarchical and potentially paternalistic structures, thereby supporting their professional autonomy. Notably, the present findings suggest that teamwork may be more challenging in some wards due to interpersonal and organisational factors. Furthermore, teamwork across units may require greater attention, as previous research in Slovakia has consistently reported the lowest proportion of positive responses among HCPs in this domain (Gurková et al., 2020; Sováriová Soósová et al., 2017). Given that teamwork is integral to hospital practice, its quality is of particular importance and should therefore be actively supported by management.

Perceived or actual risk prompted HCPs to speak up about risks but also took preventive measures to make healthcare delivery safer for patients. Therefore, the present findings highlight that participants not only regarded patients’ health and safety as their core values but also engaged proactively in efforts to preserve them. Similar patterns of proactive behaviour have been demonstrated in previous studies that highlighted responding quickly after a patient safety incident and preventing recurrence in the future (Hannawa et al., 2022; Heier et al., 2021). Even when experiencing internal conflict, participants seemed to know how to act in situations where they were concerned about patient safety. However, as Blair et al. (2022) have shown, some HCPs may not be sure of how to act in situations where they witness suboptimal care, and their responses may vary greatly, ranging from passivity to speaking up and intervening. Their perception of suboptimal care and subsequent responses may be influenced by contextual factors, hospital culture, interpersonal relationships, and related influences (Blair et al., 2022).

### 4.1 Strengths and limitations

The core strength of this research lies in its examination of HCPs’ perceptions and experiences regarding patient safety concerns using a qualitative approach, which has been underrepresented in previous studies, but holds substantial potential to provide relevant contextual information essential for developing effective intervention proposals.

It is important to acknowledge that the entire research team consisted of psychologists. Approaching the study as outsider researchers simultaneously represents a strength and a limitation, depending on the perspective taken. When viewed as a strength, this allowed the authors to bring a psychological lens to a topic that is predominantly explored in medical and nursing research. Conversely, the study could have been further strengthened by including a HCP within the research team to provide insights from the healthcare system and better shape the design of the study, development of interview questions, and analysis and interpretation of the findings.

Another limitation of this study is the subjectivity of the researchers in selecting participants, which may have influenced the final sample. The authors attempted to recruit HCPs from diverse hospital settings, departments, and specialities. Nonetheless, certain groups (e.g., professionals employed in private hospitals or in hospitals operating as joint-stock companies) were represented by only a small number of participants, and greater representation from these groups may have further enriched the findings.

The possible influence of the interviewers being outsider researchers must also be emphasised. This may have caused participants to not develop trust in the interviewer, which may have led them to withhold information relevant to the research that they would not have withheld if the interview had been conducted by someone from their organisation (e.g., a clinical psychologist or another HCP). To address this limitation, both interviewers sought to create a non-judgemental and confidential atmosphere while trying to show genuine interest in participants’ perspectives.

Given that the sample comprised HCPs across different positions, including physicians and nurses, there are potential interpretative limitations in the analysis. Firstly, focusing on a subcorpus may have resulted in findings that were more narrowly centred on a specific aspect, namely experiences of concerns for patients and related phenomena in hospital practice. In contrast, working with the full dataset could have yielded broader findings more closely related to factors influencing speaking up and HCPs’ strategies, however, given the size of the dataset, these may have been less detailed.

Furthermore, the phenomenon under investigation was not explicitly present in all interviews. From an RTA perspective, this does not diminish its analytical significance, however, it should be taken into account in the interpretation of the findings. There may be several explanations for this. For instance, participants may have referred to the phenomenon in a more implicit and ambiguous way, which may have resulted in some meanings being less visible during analysis. It is also possible that this was not the first issue that came to participants’ minds when responding to the interview questions. In addition, more active probing on this aspect during the interviews might have elicited further relevant insights.

In addition, some subthemes may have been more strongly informed by nursing perspectives, whereas others may have reflected physicians’ experiences to a greater extent. However, it is important to note that the themes nevertheless represented shared patterns of meaning across the sample as a whole. For example, issues related to night shifts were particularly prominent in nurses’ accounts, although similar experiences were also reported, to a lesser extent, by physicians. At the same time, a strength of the analysis is that, despite the mixed professional sample, findings that may be especially relevant to either medical or nursing practice were not obscured and remained visible within the data. Furthermore, the inclusion of participants from different professional roles may have enhanced the ecological validity of the findings by more closely reflecting the realities of hospital practice, where the delivery of healthcare relies on collaboration among professionals from different disciplines and positions.

### 4.2 Implications

The findings indicate that strategies and plans to improve patient safety at the global (e.g., WHO, 2021) and national levels (e.g., Ministry of Health of the Slovak Republic, 2019) may need to be supported by systematic efforts from hospital management and supervisory staff to foster a safety culture embraced at both individual and team levels. It is essential that hospital management and supervisory staff emphasise patient safety and shared patient-protection goals and support open communication, which has the potential to encourage HCPs to speak up about concerns and suggestions for improvements that are critical for patient safety (Lee S. E. et al., 2023). Speaking up could be further endorsed by cultivating psychologically safe workplaces characterised by trust, respect, and a culture focused on cooperation and innovation rather than punishments (Ito et al., 2022). A major challenge remains the power imbalance between physicians and nurses, which can be addressed during training by incorporating more shared clinical practice into both medical and nursing education, thereby fostering respectful and collaborative practice. Moreover, sharing responsibility for patients could also strengthen interprofessional collaboration and thus patient safety (Brandt et al., 2025).

## 5 Conclusion

The present study indicated that HCPs recognised risks associated with the provision of healthcare and conceptualised patient safety concerns less in terms of their causes and more in relation to ensuring patient safety and high-quality care. In this sense, participants viewed patients as their foremost priority and responsibility, and their strong professional commitment to patient care appeared to shape their emotional experiences. When concerns about patients arose, HCPs’ responses depended on the situational context. Non-acute situations allowed for careful consideration of benefits and risks for both patients and HCPs themselves, whereas acute situations required rapid action. Concurrently, participants reported several substantial barriers to the delivery of high-quality care that caused internal conflict and stemmed from ineffective collaboration and communication, exposure to unsafe healthcare, and inadequacies in the established hospital processes or infrastructure. These barriers also included a persistent imbalance between physicians and nurses, which may be rooted in the historical tradition of paternalism in medicine. The results of the study highlighted that experiences with suboptimal care motivated HCPs to protect patients by speaking up, intervening, or implementing preventive measures. The identified barriers could be addressed by strengthening a safety culture as well as promoting teamwork, shared responsibility, and open communication supported by psychological safety within hospital teams in Slovakia. Importantly, the findings of the present study may inform future research in hospital settings in other Central European countries with similar post-socialist legacies. Future research could qualitatively explore different aspects of safety culture in Central European hospitals, including hospitals in Slovakia.

## Data Availability

The datasets presented in this article are not readily available because of ethical restrictions and its sensitive nature, which could compromise participant confidentiality. Requests to access the datasets should be directed to Lucia Kupkovicova, kupkovicova7@uniba.sk.

## Appendix: interviewing guide

*Factors influencing psychological safety when speaking up about patient safety concerns*

1. What do you consider in situations when you are concerned about patient safety?
2. What influences whether you speak up in the workplace about patient safety concerns, or not?

   a. What/what factors make it easier for you to openly express your concerns?
   b. What/what factors make it difficult for you to openly express your concerns?

3. How do you feel when you are about to express your concerns?

   a. In what situations did you feel comfortable expressing your concerns?
   b. In what situations did you not feel comfortable expressing your concerns?

4. To what extent do you perceive that you can express concerns about patient safety at your workplace?

Strategies for speaking up about patient safety concerns

5. When you express a concern about patient safety, how does it usually go?

   a. How do you express concerns? What type of communication do you use?
   b. If you use any strategies to express concerns, what are they?
   c. How do you choose who to share your concerns with?
   d. Who do you usually communicate concerns to? Why?
   e. Who do you not communicate concerns to? Why?

6. Could you describe the last time you or your colleagues expressed concerns about patient safety?
7. Could you describe a situation when you wanted to express concerns about patient safety, but did not express them?

*Suggestions for strengthening psychological safety to support speaking up about patient safety*

8. What would you like to see implemented in your department to encourage open communication about patient safety concerns?
9. What changes would you like to see in your hospital to support open communication about patient safety concerns?
10. What changes would you like to see to support open communication about patient safety concerns in healthcare in general?

Final question: 11. Is there anything you would like to add on this topic?
